# Probing local innate immune responses after mucosal immunisation

**DOI:** 10.1186/1476-8518-8-5

**Published:** 2010-09-13

**Authors:** Lindsay J Hall, Simon Clare, Gordon Dougan

**Affiliations:** 1 Wellcome Trust Sanger Institute, Wellcome Trust Genome Campus, Hinxton, Cambridgeshire, CB10 1SA, UK

## Abstract

**Background:**

Intranasal immunisation is potentially a very effective route for inducing both mucosal and systemic immunity to an infectious agent.

**Methods:**

Balb/c mice were intranasally immunised with the mucosal adjuvant heat labile toxin and the *Mycobacterium tuberculosis *fusion protein Ag85B-ESAT6 and early changes in innate immune responses within local mucosal tissues were examined using flow cytometry and confocal microscopy. Antigen-specific humoral and cellular immune responses were also evaluated.

**Results:**

Intranasal immunisation induced significant changes in both number and distribution of dendritic cells, macrophages and neutrophils within the nasal-associated lymphoid tissue and cervical lymph nodes in comparison to controls as early as 5 h post immunisation. Immunisation also resulted in a rapid and transient increase in activation marker expression first in the nasal-associated lymphoid tissue, and then in the cervical lymph nodes. This heightened activation status was also apparent from the pro-inflammatory cytokine profiles of these innate populations. In addition we also showed increased expression and distribution of a number of different cell adhesion molecules early after intranasal immunisation within these lymphoid tissues. These observed early changes correlated with the induction of a T_H_1 type immune response.

**Conclusions:**

These data provide insights into the complex nature of innate immune responses induced following intranasal immunisation within the upper respiratory tract, and may help clarify the concepts and provide the tools that are needed to exploit the full potential of mucosal vaccines.

## Background

In recent years the nasal route for vaccination has emerged as an attractive mucosal route for inducing both local and systemic immunity and offers some important opportunities for the prophylaxis of many diseases. In addition to the generation of strong local mucosal immune responses within the respiratory tract, the nose can also act as an ideal inductive and effector site for immune responses at distal mucosal sites such as the lung, gut and vagina via the common mucosal immune system [1-3] The rational design of nasal vaccines for clinical use depends on the availability of information about the mechanisms that lead to a mucosal immune response after i.n. vaccination [4]. Unfortunately, despite its role in mucosal immunity, little is known about the immune system within the upper respiratory tract (URT).

The role of lymphoid tissues in respiratory tract defences includes antigen uptake, processing and consequent presentation for the induction of mucosal immune responses. In rodents this has been found to occur in the secondary organised lymphoid aggregate, called the nasal-associated lymphoid tissue (NALT), located at the floor of the nasal cavity [1,5,6]. The NALT is the first point of contact for many inhaled antigens, and consequently plays a major role in both induction and effector immune responses, which are then further amplified in the draining cervical lymph nodes (CLN) [7]. In humans, the nasopharyngeal region also contains a high density of immune competent cells similar to the NALT, most notable in the Waldeyer's ring which consists of the tonsils and adenoids [8].

In addition to the generation of adaptive immune responses, the induction of innate immunity is also crucial for vaccines to elicit potent antigen specific immune responses. However, despite i.n. immunisation emerging as one of the most promising mucosal routes for vaccine delivery, few studies have examined the innate immune populations recruited and consequently induced within the URT early after i.n. administration of antigen. The majority of studies looking at the NALT and CLN have focussed on the induction of antigen-specific T and B lymphocytes, and have therefore tended to examine later time-points [6,9-11] A greater understanding of innate immune processes, conducted by cells relatively unrestricted in antigen specificity, including, DC, MФ and neutrophils (PMN) is therefore required.

The impact of immunisation on the expression of mucosal homing receptors on circulating immune cells, as well as mucosal addressin cell adhesion molecule-1 (MAdCAM-1) expression on endothelium, has been rather well studied, particularly with regards to the gut [12,13]. Oral (intestinal) mucosal exposure to antigen seems to stimulate expression of α_4_β_7 _integrins, which together with MAdCAM-1 mediates leukocyte homing [14,15]. Previous studies have shown that within both the NALT and CLN, high endothelial venules (HEVs) utilise peripheral node adressin (PNAd)-L-selectin interactions and MAdCAM-1-α_4_β_7 _interactions for leukocyte binding, although not all HEV express MAdCAM-1 [15,16]. However, as yet, it is still unknown whether this homing of specific cells is mediated by altered cell adhesion molecule (CAM) expression after i.n. vaccination in the URT lymphoid tissues.

As already mentioned, stimulation of the innate immune system is known to have an important role in the progression of adaptive immunity. Thus, inclusion of molecules, such as adjuvants, which can trigger early innate immune responses involved in the generation of strong and protective adaptive immune responses, is crucial to vaccine effectiveness. This is why we have included *Escherichia coli *heat-labile enterotoxin (LT) as a model for a strong mucosal adjuvant in our study. LT is a well characterised adjuvant which is known to induce strong immune responses after contact with mucosal surfaces when co-administered with soluble antigens [17]. In addition to LT, we also employed the use of the *M. tuberculosis *fusion antigen, Ag85B-ESAT6. This particular protein has been used in numerous reports and has been shown to induce strong immune responses and importantly has already been used in several recent i.n. immunisation studies [18-20]. Thus the aim of this work is to contribute to our understanding of innate immune responses induced within both the NALT and CLN early after i.n. administration of antigen and the corresponding adaptive responses that these changes provoke.

## Methods

### Animals

Female Balb/c mice (5-6 weeks) from Charles River, UK were used in all animal experiments. All animals were given food and water *ad libitum*. Mice were sacrificed by cervical dislocation or exsanguination. Animal husbandry and experimental procedures were conducted according to the United Kingdom Animals (Scientific Procedures) Act 1986.

### Immunisations

For immunisations, mice were lightly anesthetised and then i.n. immunised on day 0 with 10 μg Ag85B-ESAT6 plus 1 μg LT, or PBS (naïve mice) in a volume of 15 μL/nostril. The adjuvant LT was a kind gift from Novartis, Italy and purified Ag85B-ESAT6 was obtained from the Statens Serum Institute, Denmark.

### Immunofluorescent staining

Mice were sacrificed at 5, 24 and 72 h post i.n. immunisation and their CLN and NALT removed [21]. The CLN and NALT were snap-frozen in liquid nitrogen and 6 μm sections cut. Frozen sections were stained with primary mAb as specified in Table 1. Hoechst was used as a nuclear counterstain.

**Table 1:** Antibodies used in study.

Cell Target	Target Molecule	Clone	Isotype	Conjugate	Source
CAM	PNAd	MECA 79	IgM	none	BD Biosciences
CAM	MAdCAM-1	MECA 367	IgG2a	none	BD Biosciences
CAM	VCAM-1	MVCAM.A (429)	IgG2a	none	BD Biosciences
CAM	ICAM-1	3E2	IgG1	none	BD Biosciences
DC	CD11c	HL3	IgG1	PE-Cy7 or none	BD Biosciences
PMN	Ly6G	RB6-8C5	IgG2b	PE or none	BD Biosciences
MФ	F4/80	C1:A3-1	IgG2b	none	AbD Serotec
MФ	F4/80	BM8	IgG2b	TRI-COLOR	Invitrogen
PMN	CD69	H1.2F3	IgG1	PE or FITC	BD Biosciences
DC and MФ	I-A^b ^(MHC II)	AF6-120.1	IgG2a	FITC	BD Biosciences
DC and MФ	VCAM-1	MVCAM.A (429)	IgG2a	Alexa Fluor 647	AbD Serotec
DC, MФ and PMN	IFN-γ	XMG1.2	IgG1	Alexa Fluor 700	BD Biosciences
DC, MФ and PMN	IL-10	JES5-16E3	IgG2b	APC	BD Biosciences
DC, MФ and PMN	TNF	MP6-XT22	IgG1	PE-Cy7	BD Biosciences
all cells	Hamster Ig	none	Ig	Alexa Fluor 488 or 568	Invitrogen
all cells	Rat Ig	none	Ig	Alexa Fluor 488 or 568	Invitrogen

### Flow cytometry

Single cell suspensions from the NALT and CLN of individual mice were prepared to obtain a final concentration of 5 × 10^5 ^cells/well in blocking buffer (1 × PBS/1% BSA/0.05% sodium azide/1% rat, hamster and mouse serum). To this; 50 μL of each mAb dye mix was added plus 5 μL of the amine-reactive viability dye ViViD (Invitrogen) to determine dead cells, with incubation in the dark at 4°C for 30 min [22]. The mAb used for flow cytometry are listed in Table 1. Cells were washed twice with blocking buffer and finally resuspended in 200 μL 1% paraformaldehyde. To perform flow cytometric analyses and measure relative fluorescence intensities a FACSAria cytometer and BD Diva software (Becton Dickinson) were used. For each mouse 20,000-200,000 events were recorded. The percentage of cells labelled with each mAb was calculated in comparison with cells stained with isotype control antibody. Background staining was controlled by labelled isotype controls (BD Biosciences, Invitrogen and AbD Serotec) and fluorescence-minus-one (FMO). The results represent the percentage of positively stained cells in the total cell population exceeding the background staining signal. For determination of intracellular cytokine production, cells were incubated for 6 h at 37°C with BD Activation Cocktail plus GolgiPlug or GolgiPlug alone (BD Biosciences). Cells were then washed with staining buffer and stained at 4°C with appropriate surface mAb listed in Table 1. Cells were then fixed and saponin-permeabilised (Perm/Fix solution, BD Biosciences) and incubated with cytokine mAb listed in Table 1 or isotypic controls. After 30 min cells were twice washed in permealisation buffer (BD Biosciences) and then analysed by flow cytometry as described above.

### Evaluation of antibody responses

Serum samples from mice were obtained on day 28 post immunisation and analysed for the presence of total Ig, IgG1 and IgG2a. Briefly, ELISA plates (Nunc Maxisorp) were coated overnight at 4°C with 50 μL of a 2 μg/mL solution of purified Ag85B-ESAT6 in coating buffer (0.1 M Na_2_HPO_4 _at a pH of 9) and then blocked with 3.0% BSA at room temperature for 1 h. Serum samples were diluted in 0.1% BSA starting at 1:100. Each plate contained control wells with preimmune sera, PBS (pH 7.4), or a known positive immune serum and incubated for 1 h at 37°C. Antibodies conjugated to HRP were diluted 1:1000 and plates incubated for a further 30 min at 37°C. The level of HRP associated with each well was then determined using Sigma Fast *o*-phenylenediamine dihydrochloride (50 μL per well) colorimetric substrate. The reaction was stopped after 15 min by the addition of 2.5 M H_2_SO_4_. The OD_490 _was determined, and the titre was expressed as the reciprocal of the dilution giving an OD of 0.2.

Lung and nasal lavages, performed post-mortem, were used to measure mucosal antibody responses. Lavages were performed using 1 mL of ice-cold PBS containing a cocktail of protease inhibitors (Roche), which was flushed in and out of the lungs and nasal passages with a fine-tipped Pasteur pipette inserted via the trachea. The ELISA protocol was modified as follows; lavage fluid was diluted in 0.1% BSA starting at 1:10. IgA conjugated to biotin (BD Biosciences) was used as the secondary antibody at 1:1000. To detect the biotin-conjugated antibody, 50 μL of streptavidin-HRP diluted at 1:1000 was added to each well. Plates were developed and titres measured as described above.

### Cytometric Bead Array cytokine analysis

Spleens were removed from mice 28 days post immunisation and seeded, in duplicate at a concentration of 5 × 10^5 ^cells/well. Splenocytes were stimulated with either Ag85B-ESAT6 or Concanavalin A (both at 5 μg/mL) or RPMI^+ ^medium. Plates were incubated at 37°C and 5% CO_2 _for 36-48 h. Cytokines IL-2, IL-6, IL-12, IL-10, IFN-γ and TNF-α were analysed using cytometric bead analysis flexi-kits (BD Biosciences) and assays were performed per the manufacturer's instructions. Samples were then analysed on a FACSAria flow cytometer (BD Biosciences). The limit of detection was ~ 8 pg/mL for all cytokines.

### Statistical analysis

Experimental results were plotted and analysed with Prism4 software (GraphPad Software Inc, CA, USA). Statistical significance was determined by using the Mann-Whitney *U *test or by one-way ANOVA followed by Bonferroni's multiple comparison test compared to naïve mice (i.e. 0 h).

## Results

### i.n. immunisation induces significant changes in the percentage and total cell number of innate cell populations within both the NALT and CLN

**Figure 1:**
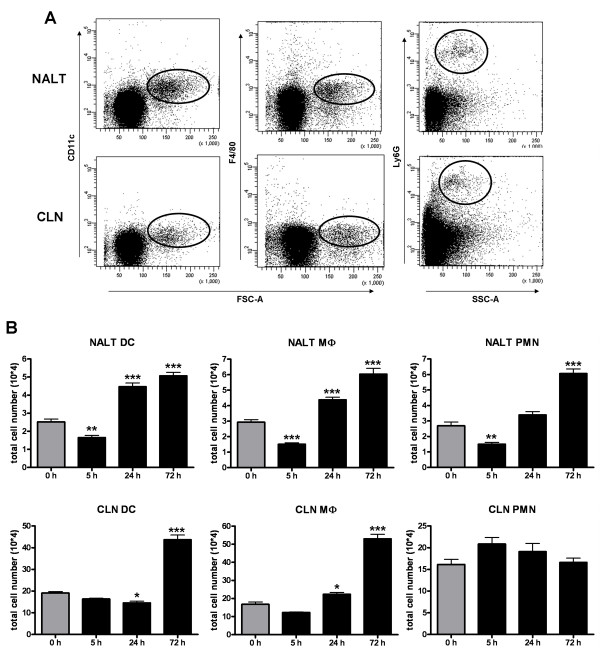
**Kinetics of innate immune cells isolated from the NALT and CLN 5, 24 and 72 h post i.n. immunisation**. Cells were isolated from NALT and CLN of Balb/c mice 5, 24 and 72 h after i.n. immunisation with 1 μg LT + 10 μg Ag85B-ESAT6, or PBS (naïve mice) and stained with flurochrome-labelled mAb and analysed by flow cytometry in which 20,000-200,000 events were recorded. **(A) **Representative dot plots showing CD11c, F4/80 and Ly6G positive populations from both the NALT and CLN of naïve mice. **(B) **Total cell number is shown for each organ. Columns represent the mean number ± SD. The * indicates significant values of p < 0.05; **, p < 0.01; ***, p < 0.001, as determined by one-way ANOVA followed by Bonferroni's multiple comparison test compared to naïve mice (i.e. 0 h).

To determine what innate cells were recruited and activated after i.n. immunisation groups of mice were i.n. immunised with either Ag85B-ESAT6 from *M. tuberculosis *mixed with *E. coli *LT adjuvant, or PBS (naïve mice) and their NALT and CLN were removed and analysed by flow cytometry (Fig. 1 and Table 2).

**Table 2:** Percentages of innate populations within the NALT and CLN after i.n. immunisation.

Tissue	Cell Type	0 h	5 h	24 h	72 h
**NALT**	**DC**	6.5 ± 0.6	5.4 ± 0.5	7.8 ± 0.8*	6.7 ± 0.5
	**MФ**	7.6 ± 0.8	4.9 ± 0.4***	7.6 ± 0.9	7.9 ± 1.1
	**PMN**	6.9 ± 1.1	4.9 ± 0.4**	5.9 ± 0.8	8.0 ± 1.2
**CLN**	**DC**	4.1 ± 0.3	4.3 ± 0.3	2.4 ± 0.2***	5.5 ± 0.6***
	**MФ**	3.6 ± 0.3	3.2 ± 0.2	3.7 ± 0.4	6.7 ± 0.7***
	**PMN**	3.5 ± 0.3	5.5 ± 0.9***	3.2 ± 0.3	2.1 ± 0.2**

Cells were isolated from NALT and CLN of Balb/c mice 5, 24 and 72 h after i.n. immunisation with PBS (naïve mice) or LT + Ag85B-ESAT6, and stained with flurochrome-labelled mAb and analysed by flow cytometry in which 20,000-200,000 events were recorded. Numbers are mean percentage of total cells ± SD. * indicates significant values of p < 0.05; **, p < 0.01; ***, p < 0.001, as determined by one-way ANOVA followed by Bonferroni's multiple comparison test compared to naïve mice (i.e. 0 h).

Kinetic analysis revealed that i.n. immunisation differentially influenced several cell populations during early time-points, and significant changes in percentage and relative cell number were evident as early as 5 h post immunisation (Fig. 1 and Table 2). For example, percentages and numbers of NALT DC (defined as CD11c^+^), MФ (F4/80^+^) and PMN (as Ly6G^+^), were significantly (p < 0.01) diminished 5 h post immunisation. However, we then observed a steady and significant (p < 0.001) increase in NALT innate cells from 24 h up until 72 h post immunisation. The percentage of CLN PMN was increased 5 h post immunisation, with a subsequent reduction apparent 72 h post immunisation (Table 2). As observed in the NALT, total MФ numbers were also significantly (p < 0.01) increased in the CLN of immunised mice from 24 h. After an initial decrease in CLN DC population at 24 h, this was followed by an impressive increase (over 2 times) when compared to naïve mice (Fig. 1B).

### Innate populations are activated shortly after i.n. immunisation within both lymphoid tissues

**Figure 2:**
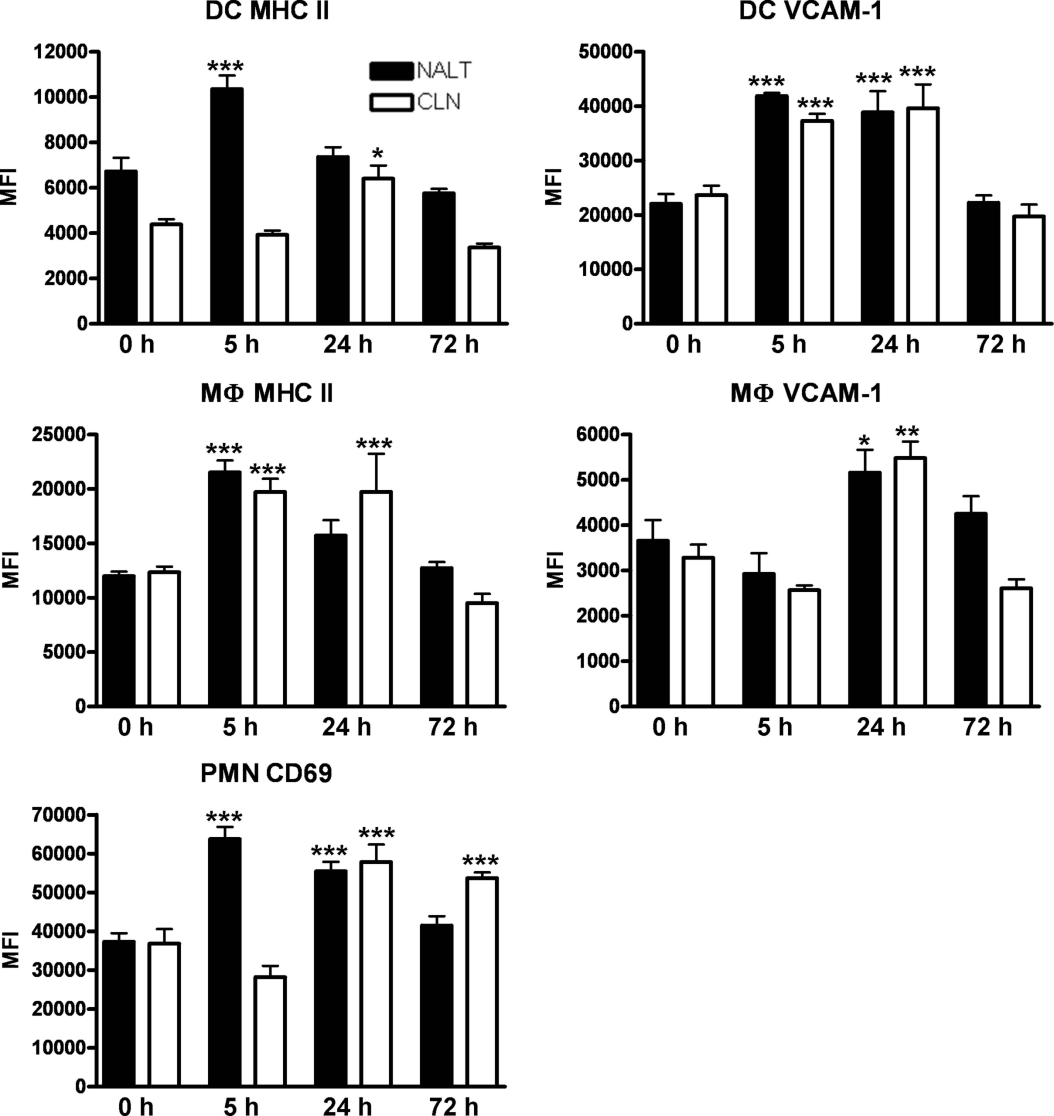
**Activation status of innate cell populations 5, 24 and 72 h after immunisation**. Isolated cells were analysed for MHC II expression, VCAM-1 expression and, CD69 expression. For analysis, gates were set on the innate subset marker positive (CD11c^+ ^or F4/80^+^) cells and the MFI of MHC II and VCAM-1 expression was determined. A gate was also set around Ly6G^+ ^cells and the level of CD69 expression was determined. Data are presented as mean MFI ± SD with the * indicating significant values of p < 0.05 and **, p < 0.01, as determined by one-way ANOVA followed by Bonferroni's multiple comparison test compared to naïve mice (i.e. 0 h).

i.n. immunisation also resulted in qualitative changes in both NALT and CLN innate populations during the first 72 h of immunisation (Fig. 2). First up-regulation of MHC II was apparent within the NALT and CLN DC and MФ populations 5 h post immunisation. Likewise, VCAM-1 fluorescence was significantly increased (p < 0.001) on DC within both tissues. Surface expression of the activation marker CD69 was increased on PMN within the NALT of immunised, compared to control mice at the 5 h time-point (Fig. 2). At 24 h we observed a significant increase (p < 0.001) in VCAM-1 expression on both DC and MФ cells within the CLN and the NALT of immunised mice. Additionally, MHC II mean fluorescent intensity (MFI) was also significantly higher (p < 0.05) on CLN DC and MФ. CD69 expression on PMN was again higher within the NALT of immunised mice and indeed we now also observed a significant increase (p < 0.001) in MFI within the CLN population (Fig. 2). By 72 h post-immunisation only the CLN PMN population had significantly higher (p < 0.001) surface expression of CD69 when compared to naïve mice (Fig. 2).

### Innate immune populations secrete a range of cytokines shortly after i.n. immunisation

**Figure 3:**
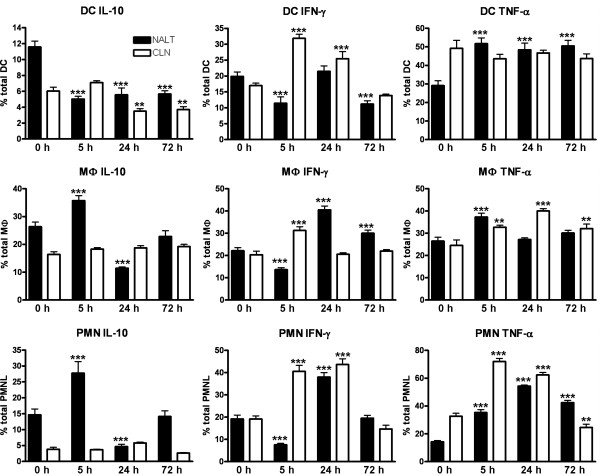
**Cytokine profile of innate immune cells shortly after i.n. immunisation**. Isolated NALT and CLN cells were stimulated for 6 h with BD Leukocyte Activation Cocktail plus GolgiPlug (or GolgiPlug alone, data not shown), stained with surface mAb to determine CD11c, F4/80 and Ly6G populations and then permeabilised and stained with anti-cytokine flurochrome-labelled mAb. Data represent percent of cytokine positive cells out of total cell population ± SD. The * indicates significant values of p < 0.05; **, p < 0.01; ***, p < 0.001, as determined by one-way ANOVA followed by Bonferroni's multiple comparison test compared to naïve mice (i.e. 0 h).

Functional aspects of the innate immune response to i.n. immunisation was addressed by *ex vivo *flow cytometric analysis of intracellular cytokine expression in cells from naïve and immunised animals (Fig. 3). Examining production of the anti-inflammatory cytokine IL-10; MФ and PMN NALT populations demonstrated an initial significant increase (p < 0.001) at 5 h post immunisation, when compared to naïve mice, followed by a decline in percentage at 24 h. In contrast, numbers of CLN MФ and PMN remained relatively stable during this 72 h period. An impressive reduction in IL-10^+ ^DC was evident in both the NALT and CLN as early as 5 h post immunisation and continued for the remainder of the study. The production of IFN-γ was also examined and we observed that all innate NALT populations were significantly diminished (p < 0.01) 5 h post immunisation. Conversely, within the CLN a dramatic increase in IFN-γ^+ ^innate cells was evident when compared to stimulated naïve mice. By 24 h percentages of NALT IFN-γ^+ ^MФ and PMN were now significantly increased (p < 0.001) when compared to naïve animals. PMN and DC IFN-γ producing CLN cells were also increased. At the final 72 h time-point NALT DC IFN-γ^+ ^percentages were significantly reduced (p < 0.001), but MФ numbers remained elevated. Analysis of another pro-inflammatory cytokine, TNF-α, revealed increased production from all innate populations throughout the study when compared to similarly stimulated naïve mice. However, TNF-α producing PMN were significantly reduced within the CLN 72 h post immunisation (Fig. 3).

### Distribution of innate cellular populations within NALT and CLN after i.n. immunisation

**Figure 4:**
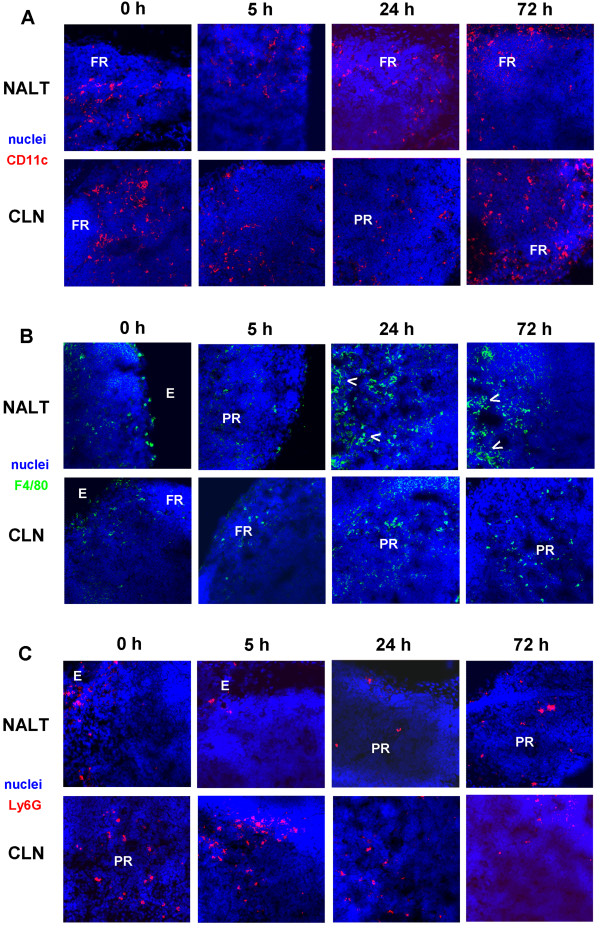
**Immunofluorescent analysis of NALT and CLN early after i.n. immunisation**. Both naïve (PBS) and immunised (LT + Ag85B-ESAT6) Balb/c mice were compared. Tissues sections from six individual mice were analysed at 5, 24 and 72 h post immunisation by confocal microscopy. For NALT and CLN, 6 μm frozen sections were stained for **(A) **CD11c (red), **(B) **F4/80 (green) or **(C) **Ly6G (red) and nuclei (blue). A representative picture for each group is shown. There was no staining using isotype control mAb (not depicted). Arrows indicate HEVs; FR, follicular regions; PR, parafollicular regions; E, edges. (Original magnification, ×28.)

Direct *in situ *visualisation of both tissues revealed a similar DC and PMN distribution to that observed within the NALT of naïve mice at 5 h post immunisation (i.e surrounding the periphery) (Fig. 4A and 4C). In contrast, DC and MФ were located more centrally (i.e. parafollicular T cell areas) within the CLN of immunised mice (Fig. 4A and 4B). From 24 h up until 72 h we observed a more wide-spread pattern of DC and PMN populations in immunised mice compared to that seen in the naïve mice within the NALT and CLN (Fig. 4A and 4C). In addition, we also observed the appearance of DC within follicular regions by 72 h. MФ present were again located centrally within both lymphoid tissues, as well as surrounding HEV in immunised mice up until the end of the study (Fig. 4B).

### CAM expression and distribution at early time-points within the NALT and CLN after i.n. immunisation

**Figure 5:**
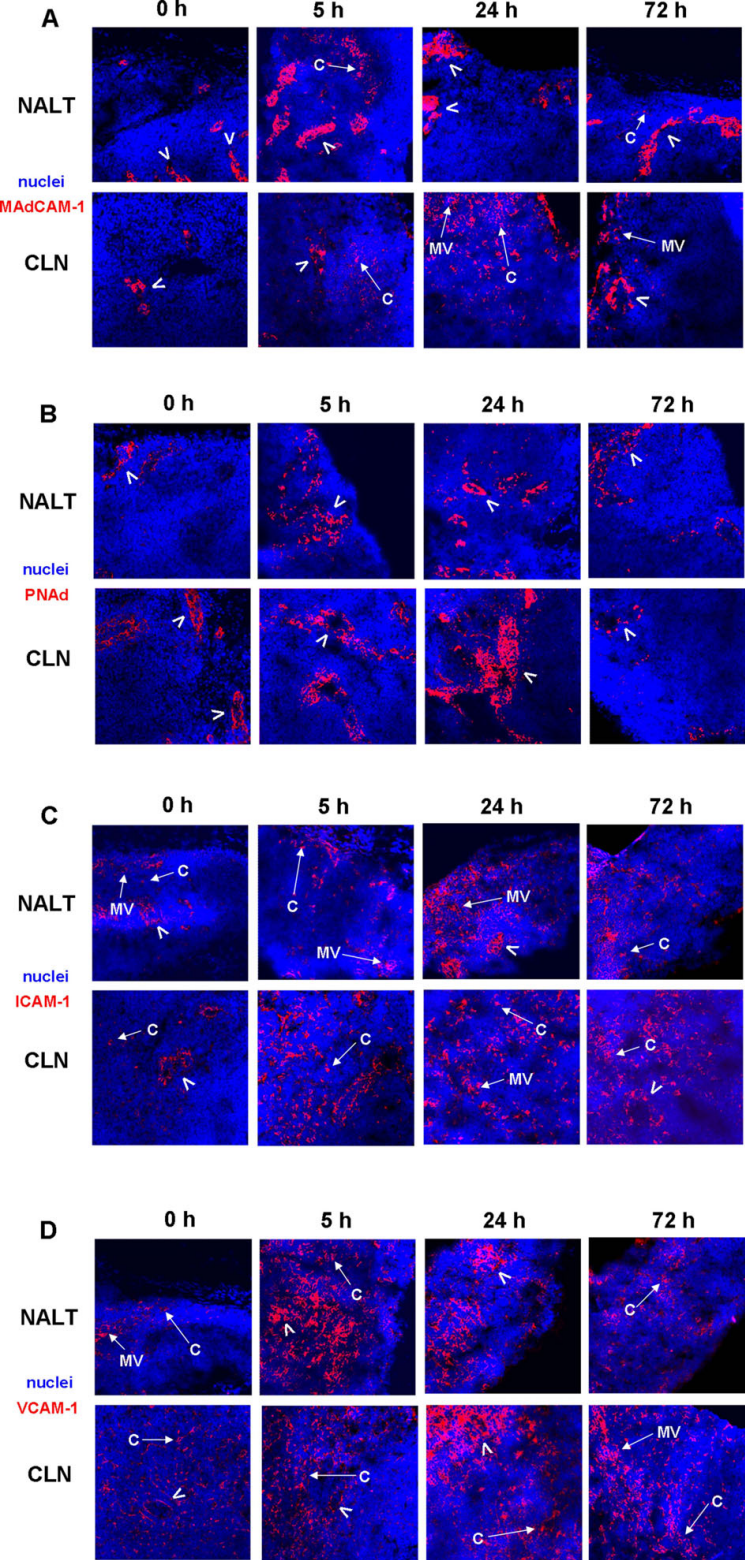
**Differential expression of CAM in the NALT and CLN early after i.n. immunisation**. Balb/c NALT and CLN were removed 5, 24 and 72 h post immunisation. Images represent 6 μm serial frozen sections stained for **(A) **MAdCAM-1, **(B) **PNAd, **(C) **ICAM-1 and **(D) **VCAM-1 (all red) and nuclei (blue). Samples were analysed by confocal microscopy. A representative picture for each group is shown for each staining. There was no staining using isotype control mAb (not depicted). Arrows indicate HEVs; C, cells; MV, micro/blood vessel. (Original magnification, ×28.)

In order to identify the CAM expression profile early after i.n. immunisation, serial frozen NALT and CLN sections of naïve and immunised (LT + Ag85B-ESAT6) mice were investigated for the distribution and expression of MAdCAM-1, PNAd, ICAM-1 and VCAM-1 at 5, 24 and 72 h. As described in previous studies, HEVs in naïve mice were found to express both MAdCAM and PNAd within their NALT and CLN (Fig. 5A and 5B) [14,15]. ICAM-1 was observed mainly in the vascular endothelium, and weak expression was also shown around micro-vessels within both lymphoid tissues. In addition, we also observed some cell surface ICAM-1 expression within the NALT (Fig. 5C). Weak diffuse VCAM-1 expression was observed in both tissues and was localised to the surface of cells and blood vessels, but not on HEVs within the NALT of naïve animals (Fig. 5D).

After i.n. immunisation, we visually observed a greater intensity of MAdCAM-1 expression, which was mainly located on the vascular endothelium of HEVs, and was higher than that seen in naïve animals in both the NALT and CLN at all time-points (Fig. 5A). In addition to venular endothelium, MAdCAM-1 was also shown on the surface of infiltrating cells in the inflamed NALT after only 5 h. All immunised mice were found to have some degree of diffuse MAdCAM-1 staining at all time-points tested within the CLN. Within both tissues we observed an increase in both PNAd expression and the number of HEVs expressing this CAM as early as 5 h post i.n. immunisation (Fig. 5B). This expression profile was still present at 24 h, although by 72 h expression levels of PNAd appeared to have reduced back to those observed in naïve animals. Greater expression of ICAM-1 was observed on HEVs, micro-vessels, and with extension to many cell surfaces within the NALT and CLN of immunised mice at all time-points when compared to naïve tissues (Fig. 5C). As with ICAM-1 expression, VCAM-1 was also observed to be more intense and widespread on HEVs, blood vessels and cells in the NALT of immunised mice (Fig. 5D). Immunisation also increased VCAM-1 expression and distribution in CLN, especially at 24 h. Expression of this CAM was also more widely distributed at 72 h within the CLN, but expression appeared lower than that observed at the 24 h time-point.

### Adaptive immune responses are induced following i.n. immunisation

**Figure 6:**
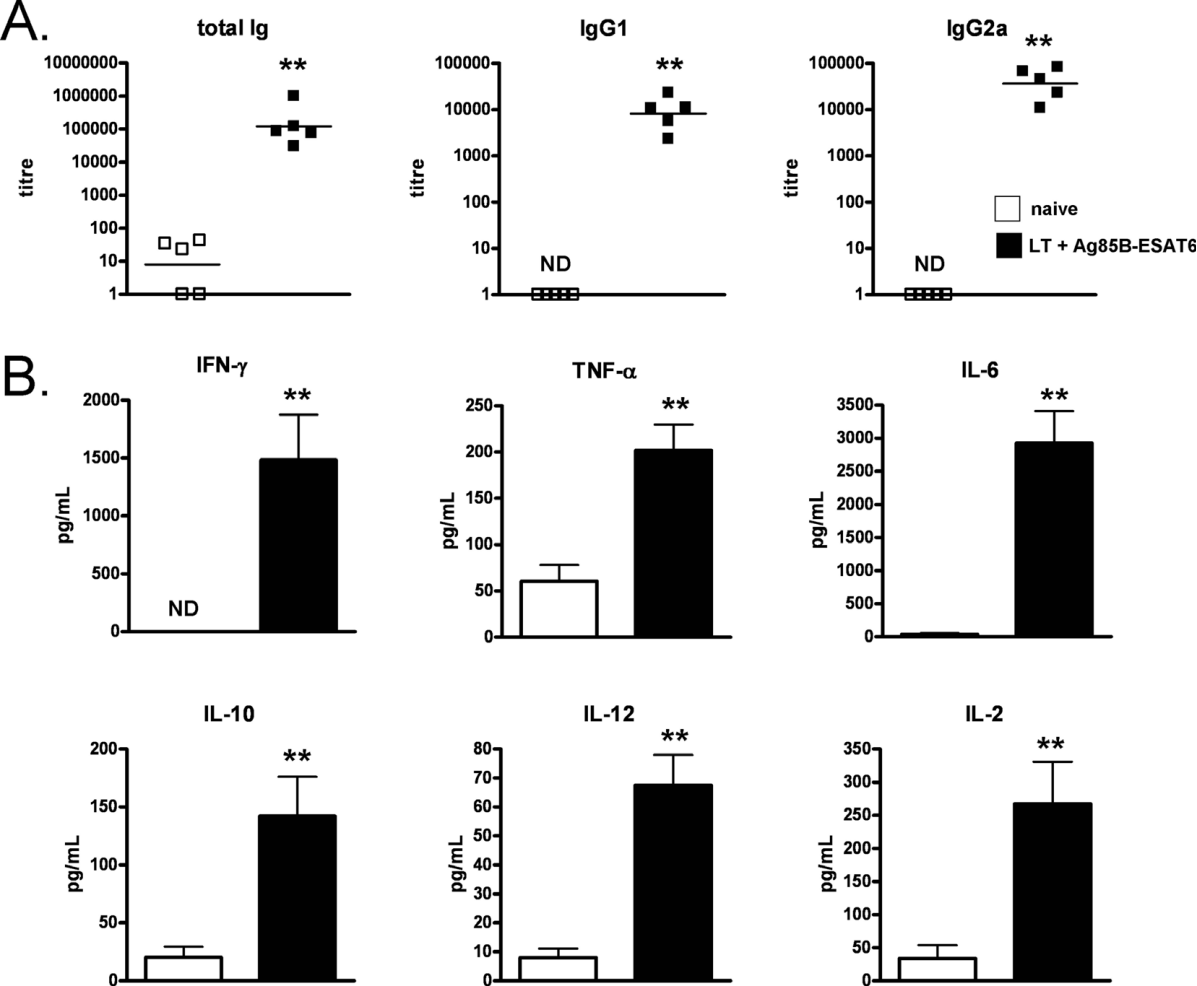
**Analysis of antigen-specific immune responses after i.n. immunisation**. Balb/c mice were immunised i.n. with 1 μg LT and 10 μg Ag85B-ESAT6, or PBS. **(A) **Mice were sacrificed 28 days post immunisation and blood collected for evaluation of primary Ag-specific Ab synthesis by ELISA. Titres are shown and reflect five individual mice per group expressed as total Ab titre using a cut-off of OD 0.2 with black bars showing the geometric mean. **(B) **Spleens were also collected for T cell assays. Cytokine responses were measured upon *in vitro *stimulation with Ag85B-ESAT6 (5 μg/mL) for 36-48 h. Columns represent the mean ± SD stimulation indices of splenocytes from 5 immunised mice. Statistical significance was determined by using the Mann-Whitney *U *test (*p < 0.05, **p < 0.01 and ***p < 0.001).

In order to confirm that the changes observed in innate populations shortly after i.n. immunisation were robust enough to induce later adaptive antigen-specific immune responses we analysed both antibody and cytokine levels 28 days post immunisation (Fig. 6). Sera collected from mice immunised with LT and Ag85B-ESAT6 was observed to have significantly higher (p < 0.001) titres of total Ig, IgG1 and IgG2a when compared to naïve mice (Fig 6A). Additionally we also examined mucosal IgA levels and observed that immunised mice had low, but significant (p < 0.05), titres: 122 ± 37 in lung and 67 ± 23 in nasal washes. Spleens were also removed from immunised mice, and these cells were stimulated with Ag85B-ESAT6 *in vitro *to assess cytokine levels (IFN-γ, TNF-α, IL-6, IL-10, IL-2 and IL-12). Significantly higher levels (p < 0.01) of each cytokine were observed in LT plus Ag85B-ESAT6 immunised compared to naïve mice (Fig. 6B).

## Discussion

The innate immune response reacts rapidly to foreign antigens with activation and migration of its cellular components [23,24]. The relevance of these individual components to host survival against pathogen infections is beyond doubt, with the protective contribution of DC, MФ, and PMN, as well as secretion of various cytokines being demonstrated in many infections [25-27]. This has lead to an appreciation in recent years that innate immunity is central to protection against infectious diseases and the induction of adaptive immune responses. However, an integrated picture of innate responses induced after mucosal vaccination has not been established. This current study goes some way to addressing this by characterising changes in defined cell populations and CAM expression in the first few hours and days following i.n. immunisation with a mucosal adjuvant and model antigen and the subsequent adaptive response generated. Hopefully this study will accentuate the importance of understanding the nature of innate immune responses at the mucosae for the design of improved vaccines.

**Figure 7:**
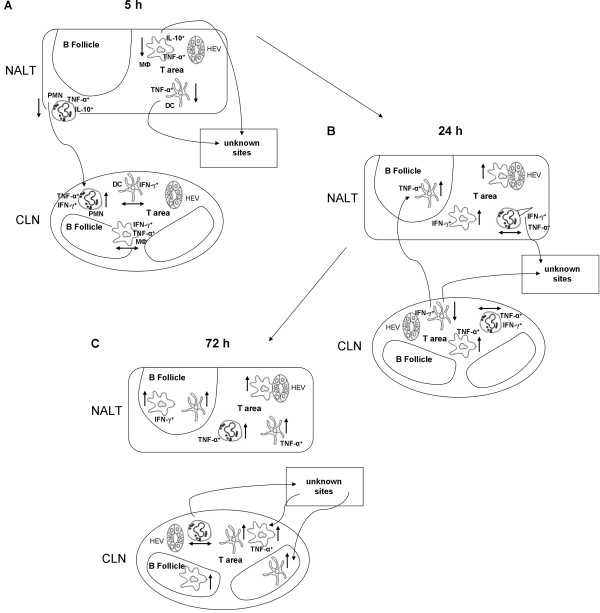
**Schematic diagram showing an overview of percentage, activation status and distribution of innate populations within the NALT and CLN early after i.n. immunisation**. Diagrams show DC, MФ and PMN within the NALT and CLN **(A) **5, **(B) **24 and, **(C) **72 h after i.n. immunisation in Balb/c mice. Short arrows beside cells indicate changes in number of cell populations; i.e. arrows pointing up show significant increases, those pointing down significant decreases and straight arrows show no change in populations when compared to naïve mice. Cytokines labelled beside cells indicate significant increases in overall percentage within corresponding innate population. Long arrows indicate the possible trafficking of cells between tissues at the various time-points.

The earliest changes in NALT cell populations detected were a rapid and significant reduction in total percentages and numbers of all cell types examined. Studies to date suggest that the NALT may have an important role in the induction of mucosal immune responses after nasal immunisation, due to the large number of unswitched naïve B and T cells, while the nasal passages, and their associated lymphocytes, may function as an effector site [28-30]. Indeed, it may be that the cell populations undergoing changes during this early 5 h time-point are in fact trafficking to this area to collect and process antigen before moving back to the inductive NALT or other lymphoid tissues. All examined cells types also appeared to be activated shortly after immunisation as indicated by increases in activation marker expression. This was further confirmed when we analysed the cytokine profile of these cells. Notably, shortly after immunisation we observed a reduction in these IL-10^+ ^cells, especially within the DC population. Several studies have shown that LT toxin exerts detectable regulatory effects on DC and MФ. After LT administration, Ag-presentation was shown to be enhanced, through increased MHC class II molecule expression and peptide presentation in APC [31]. Additionally, this mucosal adjuvant can also alter the soluble immune mediator profile of these cells; increased production of IL-6 and IL-1, but reductions in IL-12 and NO production [17,32]. These findings correlate with the concurrent increase in pro-inflammatory cytokine producing cells that are essential for initiation and maintenance of later antigen specific adaptive immune responses, especially a T_H_1 response. Indeed it appears that IFN-γ and TNF-α production via innate immune cells at these early time-points induces the observed robust T_H_1 response 28 days post immunisation. It is important to note that although activated at very early time-points, these innate cells had a similar distribution pattern when compared to naïve mice [33]. It was not until 24 h post immunisation that we observed movement of these cells to more central areas. This, coupled with impressive increases in number, along with their position in T and B cell areas, might reflect their involvement in immunologic reactions taking place in both these lymphoid tissues. We also observed that both MФ and DC had up regulated expression of VCAM-1. APC expressing this CAM are known to increase presentation of antigen to germinal centre B cells and localisation of APC within follicular areas suggests this subset of cells may be involved in the induction of the observed antigen-specific humoral immune responses generated after i.n. immunisation. From our staining, we also observed that these innate cells, especially MФ, were surrounding HEVs, possibly indicating migration from the URT through the lymphatic's to other lymphoid tissues for induction of further immune responses at remote sites. An overview of these cellular changes within the NALT and CLN, at all time-points, is shown in Fig. 7. The NALT is covered in ciliated respiratory epithelium interspersed with M-cells which enables the efficient absorption and sampling of antigens that are inhaled. Although not examined in this study, epithelial cells are another important cellular population that encounters foreign antigens and are notable sources of immunoregulatory cytokines and chemokines (including IL-10, TGF-β and CXCL8). Indeed, a previous study has shown that LT enhanced IL-6 and IL-10 secretion by intestinal epithelial cells [34]. Future studies could address the role that this cell population may play in vaccination immunity.

Another aim of this study was to examine the expression of a number of different CAM within both lymphoid tissues early after i.n. immunisation. We have shown that CAM expression of MAdCAM-1, PNAd, ICAM-1 and VCAM-1 is both increased, and more widely distributed, at all examined time-points in both the NALT and CLN of immunised mice. It has previously been demonstrated that both NALT and CLN express all these CAMs on their HEVs [14,15,35]. We also observed expression of these addressins on HEVs in naïve mice and in addition, we found that as early as 5 h post i.n. immunisation, expression of MAdCAM-1 and PNAd was further up regulated on HEVs in both tissues. This increased expression is similar to that observed in other studies after either oral immunisation or challenge in the gut mucosa [13,36-38]. It is likely that the increased MAdCAM-1 and PNAd expression in the respiratory mucosa after immunisation, possibly due to the increased local production of pro-inflammatory cytokines via innate cells, is one of the mechanisms controlling the recruitment of leukocytes to the site of inflammation [15]. We also observed that ICAM-1 and VCAM-1 expression was dramatically induced, following i.n. immunisation, on both vascular endothelium and cell surfaces indicating that leukocyte trafficking to the NALT and CLN may also depend on the interactions of α_L_β_2 _and α_4_β_1 _cells. It is interesting to note that these same molecules are involved in trafficking in the genito-urinary tract, which may explain why high levels of antigen-specific immune responses are induced in the genital tract after nasal immunisation [39]. As already discussed, VCAM-1 is essential for binding of follicular DC to B cells and for the consequent formation of germinal centres. Additionally, along with ICAM-1, these molecules enable binding to T cells to form part of the stable T cell-APC immunological synapse, which is essential for antigen presentation [40-42]. The increased expression of VCAM-1 and ICAM-1 on the surface of leukocytes indicates a possible role in early recruitment and retention of immune cells, enabling increased antigen presentation, and the consequent induction of antigen specific antibody and cytokine immune responses we observed.

## Conclusions

From our data it appears that specific stimulation of innate responses, through administration of adjuvant and antigen i.n., enables conditioning of the immune system for subsequent development of specific T_H_1 adaptive immunity. These findings may consequently help in the design and construction, via specific targeting of innate populations, of new vaccines against diseases that require this type of immune response for protection. It is hoped that this current research will ultimately provide researchers with a clearer and more informed platform for studying basic immune mechanisms, and may help provide the tools required to exploit the full potential of mucosal vaccines.

## Competing interests

The authors declare that they have no competing interests.

## Authors' contributions

LJH contributed to the design of the study, carried out all experiments and analysis and drafted the manuscript. SC participated in the design of the study, flow cytometry experiments and drafting of the manuscript. GD conceived the study, and participated in its design and helped to draft the manuscript. All authors read and approved the final manuscript.
